# Safety and Efficacy of Influenza Vaccination in Patients Receiving Immune Checkpoint Inhibitors. Systematic Review with Meta-Analysis

**DOI:** 10.3390/vaccines10081195

**Published:** 2022-07-27

**Authors:** Maria A. Lopez-Olivo, Valeria Valerio, Aliza R. Karpes Matusevich, Marianela Brizio, Michelle Kwok, Yimin Geng, Maria E. Suarez-Almazor, Ines Colmegna

**Affiliations:** 1 Department of Health Services Research, The University of Texas MD Anderson Cancer Center, 1515 Holcombe Boulevard, Unit 1444, Houston, TX 77030, USA; msalmazor@mdanderson.org; 2 The Research Institute of the McGill University Health Centre (RI-MUHC), Montreal, QC H4A 3J1, Canada; valeria.valerioguillen@affiliate.mcgill.ca (V.V.); ines.colmegna@mcgill.ca (I.C.); 3 School of Public Health, The University of Texas Health Science Center, Houston, TX 77030, USA; a1izark@gmail.com; 4 Division of Experimental Medicine, McGill University, Montreal, QC H4A 3J1, Canada; marianela.brizio@mail.mcgill.ca; 5 Department of Clinical Immunology and Allergy, McGill University Health Centre, Montreal, QC H4A 3J1, Canada; michelle.kwok2@mail.mcgill.ca; 6 Research Medical Library, The University of Texas MD Anderson Cancer Center, Houston, TX 77030, USA; ygeng@mdanderson.org; 7 Department of General Internal Medicine, The University of Texas MD Anderson Cancer Center, Houston, TX 77030, USA

**Keywords:** influenza vaccine, systematic review, meta-analysis, immune checkpoint inhibitors, cancer

## Abstract

The potential increased risk of immune-related adverse events (irAEs) post-influenza vaccine is a concern in patients receiving immune checkpoint inhibitors (ICI). We conducted a systematic review with meta-analysis of studies reporting the effects of influenza vaccination in patients with cancer during ICI treatment. We searched five electronic databases until 01/2022. Two authors independently selected studies, appraised their quality, and collected data. The primary outcome was the determination of pooled irAE rates. Secondary outcomes included determination of immunogenicity and influenza infection rates and cancer-related outcomes. Nineteen studies (26 publications, n = 4705) were included; 89.5% were observational. Vaccinated patients reported slighter lower rates of irAEs compared to unvaccinated patients (32% versus 41%, respectively). Seroprotection for influenza type A was 78%–79%, and for type B was 75%. Influenza and irAE-related death rates were similar between groups. The pooled proportion of participants reporting a laboratory-confirmed infection was 2% (95% CI 0% to 6%), and influenza-like illness was 14% (95% CI 2% to 32%). No differences were reported on the rates of laboratory-confirmed infection between vaccinated and unvaccinated patients. Longer progression-free and overall survival was also observed in vaccinated compared with unvaccinated patients. Current evidence suggests that influenza vaccination is safe in patients receiving ICIs, does not increase the risk of irAEs, and may improve survival.

## 1. Introduction

Immune checkpoint inhibitors (ICIs) are novel immunotherapy drugs that have revolutionized cancer therapy by significantly improving the survival of people living with certain malignancies [1,2,3,4,5]. These monoclonal antibodies block proteins that down-regulate immune responses (e.g., cytotoxic T-lymphocyte antigen 4 [CTLA-4] and programmed cell death 1 [PD-1]) or their ligands (e.g., programmed cell death ligand 1 [PD-L1]), resulting in activation of the immune system and enhancing recognition of tumor cells [6]. Despite the remarkable benefits of ICIs, the overactivity of the immune system can precipitate organ-specific or systemic immune-related adverse events (irAEs), which, if severe, may lead to treatment delay or discontinuation [6].

Influenza is a vaccine-preventable respiratory illness associated globally with significant morbidity and mortality [7]. During 2019–2020, influenza accounted for 18 million health care provider visits, 400,000 hospitalizations, and 22,000 deaths in the US [8]. Immunocompromised patients, either due to their underlying disease or immunosuppressive treatment, have an increased risk of influenza and its complications [9]. Specifically, influenza infection in cancer patients increases hospitalization and death rates four and ten times, respectively, compared to the general population [10]. Vaccination against influenza is safe, reduces mortality and improves infection-related outcomes among adults with cancer [11,12]. Consequently, annual influenza vaccination is recommended in this at-risk group [11].

The risk–benefit ratio of influenza vaccination in ICI-treated cancer patients is controversial [13]. Some studies suggest that influenza immunization may not protect against influenza and may overstimulate the immune system, increasing the risk of irAEs [14], whereas other studies report that it is safe and effective [15]. However, despite this controversy, influenza vaccination during ICI treatment is generally considered safe by most providers. Until now, no pooled analyses have been performed that could firmly confirm the safety of influenza vaccination in this population. Previous systematic reviews did not include all relevant studies and only presented results narratively [13,14,15].

This systematic review with meta-analysis aims to evaluate both the safety and efficacy of influenza vaccination in patients with cancer during treatment with ICIs.

## 2. Methods

### 2.1. Protocol and Registration

The systematic review was conducted following the methodological standards of Cochrane, as described in the Cochrane Handbook [16]. Results are reported according to the updated Preferred Reporting Items for Systematic Reviews and Meta-Analysis (PRISMA) statement [17]. The protocol for this review was registered in the International Prospective Register of Systematic Reviews (PROSPERO ID: CRD42020211946).

### 2.2. Eligibility Criteria

We included studies (randomized or not) evaluating the effects of the influenza vaccine in patients receiving ICIs. Studies were excluded if they reported data only on laboratory/basic science parameters, were case reports or reviews, did not include original research, were protocols or ongoing studies without results, had overlapping populations (i.e., two studies reporting on the same registry with overlapping periods), or presented data on patients who received pneumococcal and influenza vaccination, without separate data on influenza vaccination. We considered studies published as full-text or in abstract format.

### 2.3. Information Sources

A research librarian searched MEDLINE (through Ovid), EMBASE (through Ovid), Web of Science, and Cochrane Library from inception through to 21 January 2022. Unpublished records were searched through ClinicalTrials.gov (accessed on 22 April 2022). Additionally, reference lists from reviews on the topic and identified clinical trials were searched for possible references not otherwise found.

### 2.4. Search

The search strategy for MEDLINE is provided in Table S1. We used a broad search to capture all available evidence, including terms related to the influenza vaccine and vaccination, cancer, and ICIs (i.e., ipilimumab, pembrolizumab, nivolumab, atezolizumab, durvalumab, avelumab, and cemiplimab). No restrictions (i.e., language, date, or other) were imposed on the search strategy. Results were compiled using EndNoteX9 (Clarivate, London, UK).

### 2.5. Study Selection

Two pairs of authors (VV and MG, AM and MAL-O) screened all citations by titles and abstracts using the web app DistillerSR Version 2.35 (Evidence Partners, Ottawa, ON, Canada). Relevant citations were subjected to full-text assessment. Reasons for exclusion of the ineligible studies were independently recorded and disagreements were resolved through discussion, or when needed, a third author was consulted (MLO).

### 2.6. Data Collection Process

Three authors independently collected the data (VV, MK, MAL-O) and a fourth author cross-checked the data (AM). We used a Microsoft Excel spreadsheet to collect study characteristics and outcome data from the included studies. If more than one publication reported on the same study, the most recent results were used.

### 2.7. Data Items

Data collected included: (i) study characteristics (author, year of publication, country, design, number of centres, follow up period, and funding), (ii) participants’ characteristics (age, sex, and inclusion and exclusion criteria), (iii) intervention characteristics (description of the intervention, description of the control group, and concomitant medications), and (iv) outcome data (number of events and number of participants per treatment group for dichotomous outcomes, mean and standard deviation, and the number of participants per treatment group for continuous outcomes). Our primary outcome was the determination of irAE rates. Additional outcomes collected included immunogenicity (i.e., seroprotection and seroconversion rates), cases of influenza (i.e., influenza-like illness and laboratory-confirmed infection) and cancer-related outcomes (e.g., overall survival, progression-free survival, ICI treatment discontinuation, and death rates).

### 2.8. Risk of Bias in Individual Studies

Two authors (VV and MK) independently assessed the risk of bias for each study using the Newcastle Ottawa Scale (NOS) for observational studies. Discrepancies were resolved by consensus. The NOS is a validated tool that uses a scoring system to judge the selection process of the study groups (up to 4 points), the comparability of the groups (up to 2 points), and the ascertainment of exposure and outcome in the studies (up to 3 points). A maximum score of 9 points can be obtained; higher score indicates lower level of bias.

### 2.9. Summary Measures

We calculated proportions with their corresponding 95% confidence intervals (CI) for studies providing data on vaccinated patients. For controlled studies, dichotomous data were analyzed as risk ratios (RR) with their corresponding 95% CI.

### 2.10. Synthesis of Results

*Eligibility for synthesis*. We summarised data in a meta-analysis if two or more studies reported on the same outcome.

*Preparing for synthesis*. A random-effects model was used to pool studies. To pool proportion rates, we used the Freeman–Tukey arcsine transformation to stabilize variances and conduct a meta-analysis using inverse variance weights. The resulting estimates and CI boundaries were back-transformed into proportions. For relative risks, we used the Mantel–Haenszel approach. Data were analyzed as provided by authors; no attempts were made to contact the study authors. When a study had more than one follow-up time point, we used data from the longest follow-up available.

*Statistical and synthesis methods*. All statistical tests performed were 2-sided and considered a *p*-value of less than 0.05 as statistically significant. Data analyses were conducted using Review Manager software (version 5.4, Cochrane Collaboration, London, UK).

*Methods to explore heterogeneity*. We tested for heterogeneity with the chi-squared test and quantified it using the I^2^ statistic, with a value of 50% or greater considered to represent substantial heterogeneity. Subgroup analyses were performed to investigate whether study design or characteristics of the study participants could explain the heterogeneity observed.

### 2.11. Risk of Bias across Studies

Publication bias was assessed and quantified using funnel plots and Egger’s test if more than 10 studies reported on the primary outcome.

### 2.12. Certainty Assessment

A summary of findings table was created following the GRADE approach to rate the quality of the evidence for the primary outcome [18].

## 3. Results

### 3.1. Study Selection

**Figure 1:**
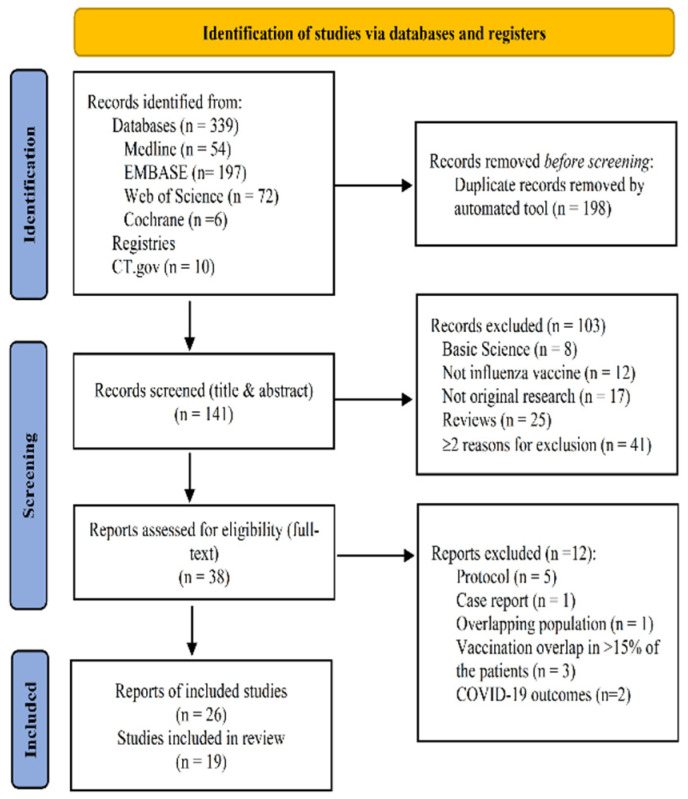
Diagram of study selection. CT.gov, ClinicalTrials.gov (accessed on 22 April 2022).

*Flow of studies*. Our research strategy identified 339 citations (Figure 1), and after de-duplication, we screened the titles and abstracts of 141 unique citations. Of these, 38 publications were considered relevant to our study and their full text was retrieved. After full-text review, 26 publications, 19 studies were found to be eligible and were included for analysis [9,19,20,21,22,23,24,25,26,27,28,29,30,31,32,33,34,35,36,37,38,39,40,41,42,43].

*Excluded studies*. Five studies were ongoing and were excluded (ClinicalTrials.gov (accessed on 22 April 2022) identifier: NCT04355806, NCT03061955, NCT03590808, NCT04697576, NCT05116917). In addition, we excluded a case report describing Guillain–Barre syndrome post-influenza vaccination [44]. Gatti et al., 2021 [45], Weber et al., 2012 [5], and Wuff-Burchfield et al., 2020 [46] were excluded due to more than 15% of patients receiving influenza vaccinations simultaneously with other vaccinations (pneumococcal and/or tetanus). Shenk et al., 2017 [47] were excluded due to possible overlapping population with Failing et al., 2020 [30]. Two reports, Bersanelli et al., 2020 and Buti et al., 2020 were excluded due to only reporting COVID-19 outcomes [48,49].

### 3.2. Study Characteristics

Table 1 provides the characteristics of the included studies. All studies were observational (11 retrospective cohorts, 1 prospective cohort, 1 case-control, 4 case series) except for two uncontrolled trials [32,35]. Ten studies were conducted in US centers, eight in European institutions, and one in South Korea. Two publications [19,20] using the same registry reported different outcomes: one provided vaccination rates in all patients reporting any type of irAEs, and the other focused only on patients reporting immune-mediated myositis. Due to the possibility of overlapping population, we considered both publications as one study.

### 3.3. Participants’ Characteristics

Table 2 shows the characteristics of the participants and the interventions reported in the included studies. A total of 4705 participants were analyzed; of these, 2108 were vaccinated. Mean age reported ranged from 54 to 67 years, and the percent of males ranged from 42% to 83%. There were different ICIs considered for inclusion, which, in all studies, were used for treating solid tumors. Vaccine administration timing also varied; six studies did not report details [22,31,33,39,42,50]. In two uncontrolled trials, the vaccine was administered on their first ICI dose on day 1 [32,35], while in the remaining studies, vaccination occurred during ICI therapy, or 7 days to 6 months before starting ICI therapy. Half of the studies reported the use of trivalent (two type A viruses, H1N1 and H3N2, and one type B virus, B/Brisbane) or a quadrivalent inactivated virus vaccine (two type A viruses, H1N1 and H3N2, and two type B viruses, B/Brisbane, and B/Phuker). One study reported 10% of the participants in the vaccination group (45/429) receiving pneumococcal or tetanus vaccination during the study [24].

### 3.4. Risk of Bias within Studies

Table S2 shows the assessment for each risk-of-bias item. In general, scores were low, given that most studies were observational and eight were published only as abstracts [28,29,31,33,38,39,42,50] (scores ranged from 3 to 9 out of a total of 9 possible). Fourteen studies were judged to have a high risk of selection bias, given that the patients were selected with specific cancer types and/or ICI, but did not include all types of ICIs or cancers [26,28,29,30,31,32,33,35,37,38,39,42,43,50]. Only two studies included all ICIs and did not exclude based on the cancer type or Eastern Cooperative Oncology Group (ECOG) performance status [20,22]. Six studies did not include a comparison group and were not evaluated for the comparability domain [9,26,28,32,33,35]. Eleven studies were judged to have a high risk of outcome bias, because it was unclear what length of time was used for the follow-up, or because follow-up time was not long enough for outcomes to occur [9,28,29,32,33,35,37,38,39,42,50].

### 3.5. Proportion of irAEs

**Figure 2:**
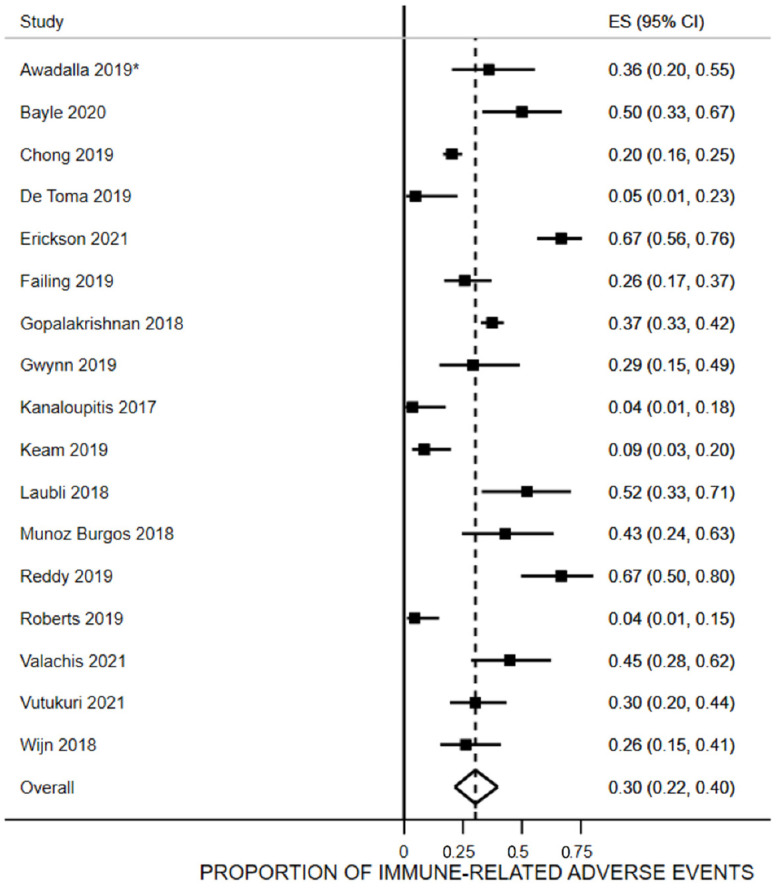
Proportion of patients receiving immune checkpoint inhibitors who were vaccinated and reported an immune-related adverse event. * Awadalla et al., 2019 reported rates of any type of irAEs in patients who already had myocarditis due to the immune checkpoint inhibitor.

Seventeen studies evaluated this outcome [9,20,26,28,29,30,31,32,33,35,37,38,39,41,42,43,50] (Figure 2). Thirty percent of the vaccinated people reported an irAE after vaccination (95% CI 22% to 40%, n = 1334, I^2^ = 90.6%). The irAEs reported in 12 of the 17 studies are shown in Table S3. The most common irAEs in the vaccinated participants were gastric (e.g., hepatitis, colitis, gastritis, transaminitis, and pancreatitis; 17 studies, range 0% to 22.2%), endocrine (e.g., hypophysitis, thyroiditis, adrenal insufficiency, hypo and hyperglycemia, diabetes, and hypothyroidism; 13 studies, range 0% to 38.9%), pulmonary (e.g., pneumonitis; 10 studies, range 2% to 25%), cutaneous (e.g., skin toxicity, dermatitis, psoriasis, or rash; 8 studies, range 0% to 68%) and rheumatic (e.g., arthritis and myalgia; 7 studies, range 2% to 13%).

**Figure 3:**
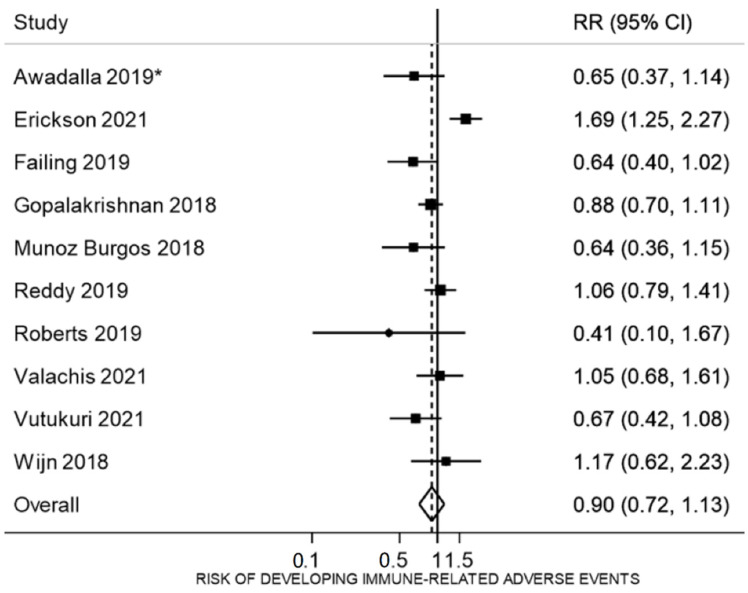
Risk of developing an immune-related adverse event in patients receiving immune checkpoint inhibitors who were vaccinated compared with those who were not vaccinated. * Awadalla et al., 2019 reported rates of any type of irAEs in patients who already had myocarditis due to the immune checkpoint inhibitor.

Ten studies compared the risk of developing any irAEs (regardless of severity) between vaccinated and unvaccinated patients [20,29,30,31,38,39,41,42,43,50] (Figure 3). The irAEs rates reported in the vaccinated group were slighter lower (32%) compared to those reported in the unvaccinated group (41%), however, the difference was not significant (RR 0.90, 95% CI 0.72 to 1.1, n = 2485, I^2^ = 64.8%). In one case-control study [19], the odds ratio of vaccination among patients with irAEs compared with those not reporting an irAE was 0.54 (95% 0.34 to 0.86, n = 540).

Median time from vaccination to irAEs was reported in four studies [26,32,36,43]. The shortest median time was 37 days (range 14 to 60) and the longest was 3.2 months (range 0 to 10.6) (Weighted median time was 88.4 ± 189.2 days). The mean difference between the vaccinated and unvaccinated groups in one study was 11.1 days (95% CI -38.6 to 16.5, n = 127) [43].

### 3.6. Immunogenicity

Seroprotection [hemagglutination inhibition (HI) titer of 1:40 which is considered an immunologic correlate corresponding to a 50% reduction in the risk of contracting influenza in adults] for influenza type A was reported by three studies [9,35,36]. Rates were 79% for H1N1 (95% CI 65% to 90%, n = 93, I^2^ = 52.9%) and 78% for H3N2 (95% CI 61% to 92%, n = 93, I^2^ = 71.3%). Seroprotection for influenza type B was reported by two studies [35,36]. Pooled rate was 75% (95% CI 64% to 85%, n = 69, I^2^ = 25%). One study reported seroconversion in 57% of vaccinated patients (i.e., an increase in antibody titers from < 1:10 to >1:40 or a >4-fold increase from a prevaccination titer of more than 1:10) [35].

### 3.7. Influenza Infection Rates

**Figure 4:**
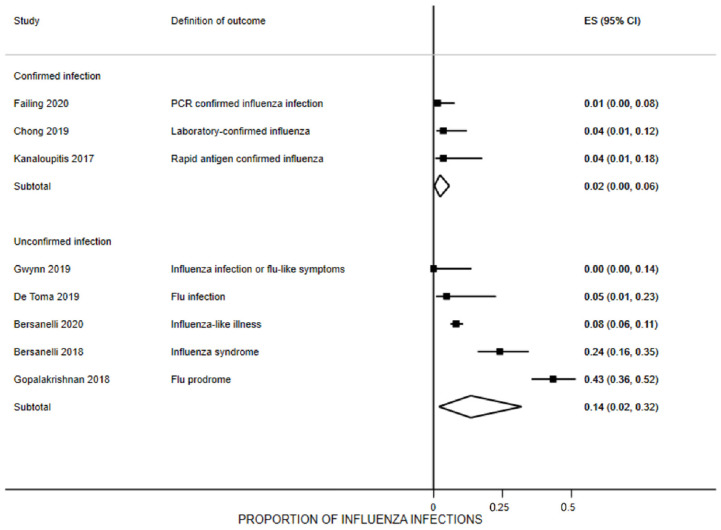
Proportion of patients receiving immune checkpoint inhibitors with influenza infection (confirmed or unconfirmed) after vaccination. For Bersanelli 2020 [23,24,25], numbers account only for patients without COVID infection.

Eight studies provided data on this outcome [22,23,26,28,30,31,32,33]. After receiving the vaccine 7 days to 6 months prior to or during ICI treatment, the pooled proportion of participants reporting an influenza-positive laboratory test was 2% (95% CI 0% to 6%, three studies [27,30,33], n = 154, I^2^ = 0%) (Figure 4). The proportion of participants reporting a laboratory-confirmed infection ranged from 1% to 4%. Four studies included control group data on this outcome [22,23,30,31]. This difference was not observed in the only controlled study where influenza was confirmed with laboratory methods [30].

**Figure 5:**
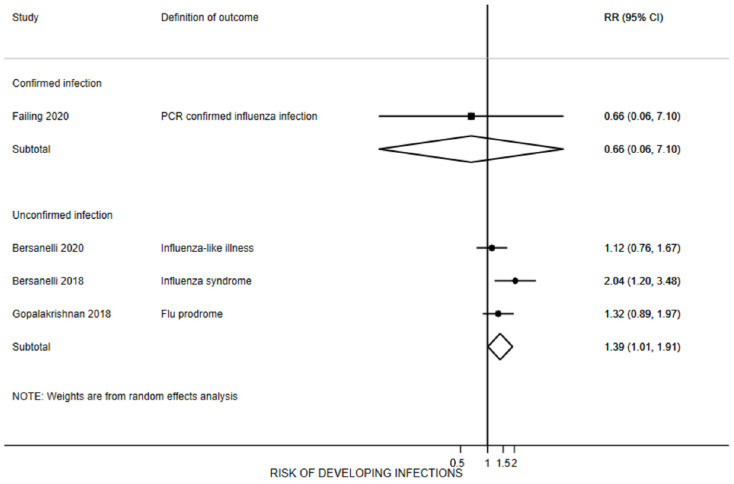
Risk of developing an influenza infection in patients receiving immune checkpoint inhibitors after vaccination compared with unvaccinated patients.

Five studies reported the proportion of participants with influenza-like symptoms without laboratory confirmation of infection [22,23,28,31,32] (Figure 5). The pool rate of patients reporting symptoms was 14% (95% CI 2% to 32%; n = 841, I^2^ = 94.5%). Although only bordering on statistically significant, vaccinated patients were 1.4 times more likely to develop influenza-like symptoms compared to unvaccinated patients (95% CI 1.0 to 1.9, n = 1846, I^2^ = 16%). Sixteen percent of those receiving the vaccine reported influenza symptoms, compared to 10% of the unvaccinated participants, with an absolute risk of 6% (95% CI -3% to 16%). The number needed to harm was 26 (95% CI 11 to 976); that is, the number of people that needed to be vaccinated in order for one person to have influenza-like illness.

### 3.8. Cancer-Related Outcomes

Five studies reported data [22,29,31,36,41]. Among vaccinated patients, the median overall survival ranged from 15.3 to 73.5 months. Two studies reported longer progression-free survival for vaccinated patients compared with unvaccinated patients (pooled HR 0.67, 95% CI 0.52 to 0.87; n = 479, I^2^ = 0%) [29,41]. In another study of 300 patients [22], the disease control rate (defined as the rate of stable diseases, partial and complete responses) for vaccinated people age 71 or above was higher than for unvaccinated patients of the same age group (published OR 2.8, 95% CI 1.0 to 7.8). Three studies reported data on overall survival, which was longer in vaccinated participants than in those unvaccinated (pooled HR 0.78, 95% CI 0.62 to 0.99; n = 779, I^2^ = 0%) [22,29,41].

ICI treatment discontinuation was reported in five studies [29,30,38,43,50]. No statistically significant differences were observed between vaccinated and unvaccinated patients (RR 1.2, 95% CI 0.74 to 1.8, n = 516, I^2^ = 65%. The median time to treatment failure reported in one study for vaccinated patients receiving ICI for non-small-cell lung cancer was 10.2 months (95% CI 6.8 to 13.6) [22].

Six studies provided data on death rates [22,25,28,30,41,43]. Influenza-related deaths were reported in two studies [22,25], irAE-related deaths in two studies [28,41], and cancer-related deaths in another two studies. Overall, death rates were similar between groups, except for cancer-related deaths, among which patients in the vaccinated group had higher mortality rates (RR for influenza-related 0.18, 95% CI 0.01 to 3.8, n = 400, I^2^ = 0%; RR for irAE-related 1.6, 95% CI 0.08 to 32.1; and RR for cancer-related 1.6, 95 CI 1.0 to 2.6).

### 3.9. Reporting Biases and Certainty of Evidence

The reporting bias assessment was performed in the primary outcome (i.e., patients receiving ICIs who were vaccinated and reported an immune-related adverse event). There was no evidence of small-study effects (Egger test *p* = 0.55) in the funnel plot (Figure S1). Table S4 shows the certainty assessment.

## 4. Discussion

In this systematic review with meta-analyses, we evaluated the risk of irAEs post-influenza vaccine. We found that the rates of irAEs were similar between vaccinated and unvaccinated patients, and the most frequently reported events were endocrine events, pneumonitis, rash, colitis, and arthritis. These data indicate that influenza vaccination does not substantially increase risk of irAEs and may be associated with lower laboratory-confirmed infections in cancer patients treated with ICIs.

Our meta-analysis found seroprotection and seroconversion rates similar to those observed in a low-risk target population (60% to 100%) [52]. Further, the proportion of participants reporting an influenza infection after vaccination differed between those studies reporting infection without laboratory confirmation and those with laboratory confirmation, with lower rates observed for those in the laboratory-confirmed group. When compared with unvaccinated patients, although there was a small absolute risk increase (6%) in the vaccinated group of developing influenza-like symptoms, this was below the estimated median incidence rate of 8% for influenza in the US [53]. We also evaluated cancer-related outcomes, and observed longer survival in vaccinated compared with unvaccinated patients, with the rates of ICI treatment discontinuation similar among groups. These encouraging results indicate that influenza vaccination is relatively safe for patients and does not interfere with ICI treatment.

A previous study hypothesized that vaccination in combination with ICI could mediate infiltration of central memory T cells into the tissues leading to an enhanced immune response [36]. An alternative hypothesis is that the increased risk may result from the cross-reactivity of T cells invigorated by influenza vaccination proteins [54]. These hypotheses combined with irAE rates above 40% reported in observational studies [9,29,36,38,41,50], led other authors to summarize the data and evaluate the efficacy and safety of influenza vaccination in patients with cancer during treatment with ICIs. To date, three systematic reviews have summarized published data on the topic [13,14,15]. Two studies provided only a narrative summary without any attempt to pool results. Of these, Bersanelli et al. described nine studies in a tabular format and concluded that there was controversial evidence and additional studies were needed [13]. Desage et al. used evidence from 10 studies to determine whether influenza vaccination induced serological protection and increased irAEs [14]. The results of each included study were summarized in a paragraph without interpretation or concluding remarks. Spagnolo et al. included 10 studies, and the authors provided descriptive statistics to pool data for irAE rates without using meta-analytic methodology for binomial data [15]. Data on efficacy outcomes were not pooled. Our study is the first systematic review with meta-analysis with data from 19 studies. We summarized and pooled data on safety and efficacy outcomes, including cancer-related outcomes, which have not been summarized previously.

There are important limitations to consider. Although we used a systematic and best-practice approach to coalesce the available evidence, more research from studies at low risk of bias is warranted, given that the confidence in our estimates of effects is low due to the non-randomized nature of the studies included, their potential for high risk of bias, and the inconsistency observed. However, for ethical reasons, no randomized controlled trials have been conducted to evaluate the benefits and harms of influenza vaccination in patients receiving ICIs. Thus, our results, despite being based on uncontrolled trials and observational studies, further the understanding of clinical outcomes in the absence of randomized trials [18]. In assessing survival, there are concerns regarding the ability to isolate the effects of vaccination from other factors that impact survival in patients receiving ICI treatment, given the absence of information on the distribution of key factors by vaccination status, such as tumor type, gender, and clinical co-factors. Differences in cancer control and progression may exist based on age for cancer patients treated with ICIs, as reported in one study [22]. We note that the mean age represented in the meta-analysis ranged from 54–67 years. Therefore, the examined studies included patients who were of relatively young age. Future studies should aim to determine the association of influenza vaccination on various clinical outcomes in elderly cancer patients.

In conclusion, the described findings provide encouraging evidence that influenza vaccination is safe in patients receiving ICI. The incidence of irAEs was similar regardless of vaccination status. Regarding efficacy, although seroprotection rates were similar to those observed in the cancer population not receiving ICI, and the data also support an improvement in survival in vaccinated patients, future larger studies of high quality are needed to corroborate the efficacy of influenza vaccination in lowering the incidence of laboratory-confirmed infections in patients with cancer receiving ICI.

## Figures and Tables

**Table 1:** Characteristics of the included studies.

Author, Year	Publication Type	Country	# of Centres	Design	Sample Size ^e^	Recruitment Period	Follow-Up	Primary Outcome	Secondary Outcomes	Funding
Awadalla 2019 [19,20] ^a^	Full-text	USA, MA	16 for cases, 1 for controls	Case-control	641 ^a^	02/2011–06/2017 and 11/2013–10/2018	290 days for controls, 175 for cases ^b^	Vaccine rates	MACE rates	None
Bayle 2020 [9]	Full-text	France	1	Case series	30	2018–2019	6 months	Seroprotection rate, seroconversion	irAEs rates	None
Bersanelli 2018 [21,22]	Full-text	Italy	21	Retrospective cohort	300	11/2016–05/2017	31 months ^b^	Influenza syndrome rates	Lethality rates, cancer outcomes ^c^	NR ^d^
Bersanelli 2021 [23,24,25]	Full-text	Italy	82	Prospective cohort	1188	10/2019–01/2020	16 months	Influenza syndrome rates, COVID-19 rates	Lethality rates, cancer outcomes ^c^	FICOG
Chong 2019 [26,27]	Full-text	USA, NY	1	Case series	370	09/2014–03/2018	512 days ^b^	irAEs rates	Infection rates	NIH
De Toma 2019 [28]	Abstract	Italy	1	Case series	75	10/2018–01/2019	NR	irAEs rates, lethality rates	Influenza syndrome rates	NR
Erickson 2021 [29]	Abstract	USA, UT	1	Retrospective cohort	176	2013–2018	NR	irAEs rates, PFS, overall survival	ICI treatment discontinuation	
Failing 2019 [30] ^a^	Full-text	USA, MN	1	Retrospective cohort	162	09/2014–08/2017	17.1 months ^b^	irAEs rates	Influenza syndrome, ICI treatment discontinuation	NR ^d^
Gopalakrishnan 2018 [31]	Abstract	USA, TN	1	Retrospective cohort	534	2010–2017	NR	Cancer outcomes ^c^	Influenza syndrome rates, lethality rates	NR
Gwynn 2019 [32]	Full-text	USA, GA	1	Uncontrolled trial	24	10/2017–12/2017	60 days	Influenza syndrome rates, irAEs rates	Cytokine levels	None
Kanaloupitis 2017 [33]	Abstract	USA, IL	NR	Case series	28	NR	90 days or more	Immunoglobulin levels, infection	Hospitalizations, irAEs rates	NR
Keam 2019 [34,35]	Full-text	South Korea	2	Uncontrolled trial	136	09/2018–11/2018	6 months	Seroprotection rates, seroconversion	irAEs rates	GC Pharma, Seoul National University Hospital Research Fund
Laubli 2018 [36,37,40]	Full-text	Switzerland	2	Retrospective cohort	34	10/2015–11/2015	60 days (37.5 months for overall survival)	Cytokine levels Seroprotection rate, seroconversion	irAEs rates, radiographic and clinical response	Schoenmakers Foundation, Goldschmidt-Jacobson Foundation, Swiss National Foundation
Munoz Burgos 2018 [50]	Abstract	Spain	NR	Retrospective cohort	42	10/2017–01/2018	NR	irAEs rates	ICI treatment discontinuation	NR
Reddy 2019 [38]	Abstract	USA, MI	NR	Retrospective cohort	117	2014–2019	NR	irAEs rates	ICI treatment discontinuation	NR
Roberts 2019 [39]	Abstract	USA, MA	1	Retrospective cohort	285	01/2014–05/2018	NR	irAEs rates	NA	NR
Valachis 2021 [41]	Full-text	Sweden	3	Retrospective cohort	303	01/2016–05/2019	15 months ^b^	PFS, overall survival	irAEs rates	None
Vutukuri 2021 [42]	Abstract	USA, LA	1	Retrospective cohort	133	08/2015–08/2019	NR	irAEs rates	NA	NR
Wijn 2018 [43]	Full-text	Netherlands	1	Retrospective cohort	127	09/2015–01/2016 and 09/2016–01/2017	107 days for cases, 118 days for controls	irAEs rates	ICI treatment discontinuation Tumor response, deaths	NR

ICI, immune checkpoint inhibitors; irAEs, immune-related adverse events; FICOG, Federation of Italian Cooperative Oncology Groups; MACE, major adverse cardiovascular events (death, cardiac arrest, cardiogenic shock, hemodynamically significant complete heart block); NA, not applicable; NIH, National Institutes of Health; NR, not reported; PFS, progression free survival; VAERS, Vaccine Adverse Event Reporting System (co-managed by the Centers for Disease Control and Prevention and the US Food and Drug Administration); VigiBase, World Health Organization’s global Individual Case Safety Report database. ^a^ Potential overlap of study population (Massachusetts General Hospital). Allen et al. (2019) [19] reports on patients with any irAEs and Awadalla et al. (2019) [19] reports on patients with myocarditis only. Allen et al. (2019) [19] has 540 patients with ICI; Awadalla et al. (2019) [19] has 101 cases with myocarditis and 201 ‘controls’ without myocarditis receiving ICI. We assumed that the 201 controls could include subjects in the abstract of Allen et al. (2019) [19]. ^b^ Median follow-up time. ^c^ Lethality rates included influenza-related deaths, influenza-relapse rates, hospitalization due to influenza illness, bacterial superinfections, and influenza syndrome duration. Cancer outcomes included objective response rate, disease control rate, time to treatment failure, and median overall survival. ^d^ Multiple conflicts of interest disclosed. ^e^ Total number of patients reported in the publication regardless of the treatment received.

**Table 2:** Characteristics of the participants in the included studies.

Study	Age, Mean Years (SD) ^a^	Males	ICIs Considered	Cancer Type	Vaccine Timing	Cases		Controls	
						Sample	Description	Sample	Description
Case-Control									
Awadalla 2019 [19,20]	65 (15.6) ^b^	72.0%	Ipilimumab, pembrolizumab, nivolumab, atezolizumab, durvalumab, avelumab, or combination	Advanced solid tumors including melanoma, NSCLC, SCCHN	Anytime from 6 months prior to ICI to receiving the vaccine during ICI therapy	151	irAEs [19]	389	No irAEs [19]
						101	Myocarditis [20]	201	No Myocarditis [20]
Case series and uncontrolled trials (prospective studies with 1 group)									
						Intervention group			
						Sample	Description		
Bayle 2020 [9]	63 (7.6)	83.0%	Nivolumab, pembrolizumab, atezolizumab	NSCLC, urothelial	7 (±2) days after the last administration of ICI	30	1 standard dose of the French National Health authorities-approved subcutaneous vaccine		
Chong 2019 [26,27]	63 (13.8)	54.0%	Ipilimumab, pembrolizumab, nivolumab or combination	Lung, melanoma, others (NS)	2 months before or after ICI administration ^c^	370	Trivalent or quadrivalent vaccines ^d^ at high or standard doses		
De Toma 2019 [28]	NR	NR	Pembrolizumab, atelozolizumab, nivolumab, durvalumab	NSCLC	Before or within 30 days after ICI start	21	NS inactivate influenza vaccine		
Gwynn 2019 [32]	61 (11.8)	42.0%	Nivolumab, pembrolizumab, atezolizumab, avelumab, durvalumab	NSCLC, melanoma, urothelial, RCC, colon, hepatocellular, head/neck	Vaccine administered in patients with at least 1 cycle of ICI	24	0.5 mL intramuscular IIV Fluarix^®^ or Fluzone^®^ quadrivalent		
Kanaloupitis 2017 [33]	NR	NR	Anti-PD-1 (NS)	NR	NR	28	Afluria (Seqirus)		
Keam 2019 [34,35]	63 (9.0)	79.0%	Nivolumab, pembrolizumab, atezolizumab	Lung, kidney, melanoma, others ^e^	Concomitantly on day 1 of ICI	46	0.5 mL GCFLU quadrivalent pre-filled syringe injection; GC Pharma ^f^		
Prospective and retrospective cohorts (studies with 2 groups)									
						Intervention group		Control group	
						Sample	Description	Sample	Description
Bersanelli 2018 [21,22]	64.3 (8.5)	69.0%	Ipilimumab, pembrolizumab, nivolumab, atezolizumab, avelumab, combinations, or chemo-immunotherapy	NSCLC, RCC, melanoma, head/neck, urothelial, gastric, colon	NR	79	Trivalent or quadrivalent vaccines ^d^	221	No vaccine
Bersanelli 2020 [23,24,25]	65.6 (11.1)	69.9%	NS	NSCLC, RCC, melanoma, urothelial, head & neck, other (NS)	During ICI therapy	429 ^g^	Trivalent or quadrivalent vaccines	402	No vaccine
Erickson 2021 [29]	64.6 (NR)	NR	NR	Metastatic melanoma	Anytime during the observation (51% before starting ICI)	90	NS	86	No vaccine
Failing 2019 [30]	63.5 (NR)	56.2%	Pembrolizumab or combined with chemo or radiation	NSCLC, melanoma, other (NS)	Within 30 days before initiation or during ICI therapy	70	High dose trivalent, quadrivalent, or NS type vaccines	92	No vaccine
Gopalakrishnan 2018 [31]	54 (NR)	76.0%	NS	Lung, melanoma, GU, breast, lymphoma	NR	385	NS	149	No vaccine
Laubli 2018 [36,37,40]	62 (10.0)	69.6%	Nivolumab, pembrolizumab	NSCLC, RCC, melanoma	Median time from ICI initiation to vaccination was 74 days (range 4 to 457 days)	23	Trivalent intramuscular (Agrippal, Novartis) vaccine ^h^	11	Healthy controls
								40	No vaccine ^h^
Munoz Burgos 2018 [50]	64.2 (NR)	64.3%	Nivolumab, pembrolizumab	NSCLC, melanoma, RCC, head/neck, breast	NR	21	NS inactive influenza vaccine	21	No vaccine
Reddy 2019 [38]	NR	NR	Anti-PD-1 or PD-L1 (NS)	NSCLC	During ICI therapy	33	19 received quadrivalent, 13 trivalent, 1 NS	53	Not vaccinated during ICI
Roberts 2019 [39]	NR	NR	NS	NSCLC	NR	45	NS influenza vaccine	240	No vaccine
Valachis 2021 [41]	67 (13)	56.4%	Nivolumab, pembrolizumab, atezolizumab	Melanoma, NSCLC, RCC	2 months before or after ICI initiation	67	NS influenza vaccine	236	No vaccine
Vutukuri 2021 [42]	NR	NR	Pembrolizumab, nivolumab, atezolizumab, durvalumab	NS lung, melanoma	NR	53	NS influenza vaccine	80	No vaccine
Wijn 2018 [43]	62.6 (3.9)	48.0%	Nivolumab	NS advanced lung	After starting ICI or 30 days before	42	NS influenza vaccine	85	No vaccine ^i^

GU, genitourinary; ICI, immune checkpoint inhibitor; NR, not reported, NS, not specified; NSCLC, non-small cell lung cancer treatment; PD1, programmed death-1; PD-L1 programmed death ligand-1; RCC, renal cell carcinoma; SC, subcutaneous; SCCHN, squamous-cell head and neck cancer. ^a^ Median and ranges were transformed into mean and standard deviations (SDs) using previously validated methods [51]. ^b^ Data provided only for Awadalla et al. (2019) [19], not reported in Allen et al. (2019) [19]. ^c^ Cohort included patients who were ICI naïve and patients who had been on ICI more than 65 days prior vaccination. ^d^ Trivalent (two type A viruses, H1N1 and H3N2, and one type B virus, B/Brisbane), quadrivalent (adding a type B virus, B/Phuker) inactivated virus vaccine. ^e^ Bladder cancer (n = 1), adrenocortical carcinoma (n = 1), and sarcoma (n = 1), head & neck (n = 1). ^f^ 15 µg of purified viral antigen from the strains A/Singapore/GP1908/2015 IVR-180 (H1N1), A/Singapore/INFIMH-16-0019/2016IVR-186(H3N2), B/Phuket/3073/2013 (Yamagata), and B/Maryland/15/2016 NYMC BX-69A (Victoria). ^g^ 43 participants received pneumococcal and 2 tetanus vaccination together with influenza vaccine. ^h^ Influenza/A/H1N1/California/2009, Influenza/A/H3N2/Texas/2012, Influenza B/Brisbane/2008. No vaccine group included only NSCLC patients undergoing ICI. ^i^ Patients who had been vaccinated more than 30 days before receiving the first dose of ICI were included in the non-vaccine group.

## Data Availability

Data presented in this study are contained within the article and Supplementary Materials.

## Supplementary Materials

The following supporting information can be downloaded at: https://www.mdpi.com/article/10.3390/vaccines10081195/s1, Table S1: Search strategies for all sources searched; Table S2: Risk of bias within studies assessed with the New-Castle Ottawa Scale; Table S3: Frequency of reported immune related adverse events in vaccinated and unvaccinated patients; Figure S1: Funnel plot for primary outcome; Table S4: Summary of findings table.
