# Supplementary material for: Safety and Efficacy of Influenza Vaccination in Patients Receiving Immune Checkpoint Inhibitors. Systematic Review with Meta-Analysis
Source: Vaccines (Basel). 2022 Jul 27;10(8):1195. doi: 10.3390/vaccines10081195 (PMC9412390; doi:10.3390/vaccines10081195)
Supplement: Supplementary file 1 [file vaccines-10-01195-s001.zip › vaccines-1806641-supplementary.pdf]

Table S1. Search strategies for all sources searched.

Line #	**MEDLINE (Ovid). Date searched 01/21/2022**
1	(checkpoint adj3 (inhibitor* or modulator* or antibod* or block*)).ti,kf,rn.
2	(checkpoint and (inhibitor* or modulator* or antibod* or block*)).nm.
3	(immun* adj3 checkpoint adj3 (inhibitor* or modulator* or antibod* or block*)).ab.
4	(("cytotoxic T lymphocyte associated" adj3 "4") or "CTLA 4" or CTLA4).ti,kf,rn.
5	("Cytotoxic t-lymphocyte antigen" adj3 "4").ti,kf,rn.
6	("Cytotoxic t-lymphocyte antigen" or "cytotoxic T lymphocyte associated").nm.
7	(ipilimumab or "MDX CTLA 4" or Yervoy or "MDX 010" or MDX010 or "BMS-734016" or BMS734016).mp.
8	("Programmed Cell Death 1" or PD1 or "PD 1").ti,kf,rn,nm.
9	(pembrolizumab or Keytruda or Lambrolizumab or "Merck 3475" or Merck3475 or "MK 3475" or MK3475 or "Sch 900475" or Sch900475 or "HSDB 8257").mp.
10	(nivolumab or "BMS 936558" or BMS936558 or "MDX 1106" or MDX1106 or "ONO 4538" or ONO4538 or Opdivo).mp.
11	("AMP 514" or AMP514 or MEDI0680 or "MEDI 0680").mp.
12	("programmed death ligand 1" or "PD L1" or PDL1 or "B7-H1" or B7HI).ti,kf,rn,nm.
13	(atezolizumab or Tecentriq or MPDL3280A or "MPDL 3280A" or RO5541267 or RO-5541267 or RG 7446 or RG7446 or "CD274 ANTIGEN" or "CD274 protein").mp.
14	(durvalumab or imfinzi or "MEDI 4736" or MEDI4736).mp.
15	(avelumab or Bavencio or MSB0010718C or "MSB 0010718C" or MSB0010682).mp.
16	("BMS 936559" or BMS936559 or MDX1105 or "MDX 1105").mp.
17	(Cemiplimab or libtayo or REGN2810 or "REGN 2810").mp.
18	or/1-17
19	exp Neoplasms/
20	(cancer* or carcinom* or tumor* or tumour* or neoplas* or malignan* or metasta* or myeloma* or leuk?emia* or lymphoma* or sarcoma* or melanoma*).ti,ab,kf.
21	or/19-20
22	18 and 21
23	exp Influenza Vaccines/
24	((influenza* or flu or laiv) adj3 vaccin*).ti,ab,kf,rn.
25	23 or 24
26	22 and 25
	**EMBASE (Ovid). Date searched 01/21/2022**
1	(immun* adj3 checkpoint adj3 (inhibitor* or modulator* or antibod* or block*)).ti,ab,hw,kw.
2	(("cytotoxic T lymphocyte associated" adj3 "4") or "CTLA 4" or CTLA4).ti,ab,kw,hw.
3	("Cytotoxic t-lymphocyte antigen" adj3 "4").ti,ab,kw,hw.

4	(ipilimumab or "MDX CTLA 4" or Yervoy or "MDX 010" or MDX010 or "BMS-734016" or BMS734016).ti,ab,hw,kw,du,tn.
5	("Programmed Cell Death 1" or PD1 or "PD 1").ti,ab,kw,hw,du,tn.
6	(pembrolizumab or Keytruda or Lambrolizumab or "Merck 3475" or Merck3475 or "MK 3475" or MK3475 or "Sch 900475" or Sch900475 or "HSDB 8257").ti,ab,kw,hw,du,tn.
7	(nivolumab or "BMS 936558" or BMS936558 or "MDX 1106" or MDX1106 or "ONO 4538" or ONO4538 or Opdivo).ti,ab,kw,hw,du,tn.
8	("AMP 514" or AMP514 or MEDI0680 or "MEDI 0680").ti,ab,kw,hw,du,tn.
9	("programmed death ligand 1" or "PD L1" or PDL1).ti,ab,kw,hw.
10	(atezolizumab or Tecentriq or MPDL3280A or "MPDL 3280A" or RO5541267 or RO-5541267 or RG 7446 or RG7446 or "CD274 ANTIGEN" or "CD274 protein").ti,ab,kw,hw,du,tn.
11	(durvalumab or imfinzi or "MEDI 4736" or MEDI4736).ti,ab,kw,kw,du,tn.
12	(avelumab or Bavencio or MSB0010718C or "MSB 0010718C" or MSB0010682).ti,ab,kw,hw,du,tn.
13	("BMS 936559" or BMS936559 or MDX1105 or "MDX 1105").ti,ab,kw,hw,du,tn.
14	(Cemiplimab or libtayo or REGN2810 or "REGN 2810").ti,ab,kw,hw,du,tn.
15	or/1-14
16	exp malignant neoplasm/
17	(cancer* or carcinom* or tumor* or tumour* or neoplas* or malignan* or metasta* or myeloma* or leuk?emia* or lymphoma* or sarcoma* or melanoma*).ti,ab,kw.
18	16 or 17
19	15 and 18
20	exp influenza vaccine/
21	((influenza* or flu or laiv) adj3 vaccin*).ti,ab,kw.
22	20 or 21
23	19 and 22
	**Web of Science (Clarivate). Date searched 01/21/2022[a]**
1	TS= (immun* NEAR/3 checkpoint NEAR/3 (inhibitor* or modulator* or antibod* or block*))
2	TS=(("cytotoxic T lymphocyte associated" NEAR/3 "4") or "CTLA 4" or CT LA4)
3	TS=("Programmed Cell Death 1" or PD1 or "PD 1" or "programmed death l igand 1" or "PD L1" or PDL1)
4	TS=(ipilimumab or "MDX CTLA 4" or Yervoy or "MDX 010" or MDX010 or tremelimumab or ticilimumab or "CP 675 206" or "CP 675206" or CP67 520 or pembrolizumab or Keytruda or Lambrolizumab or "Merck 3475" or Merck3475 or "MK 3475" or MK3475 or "Sch 900475" or Sch900475)
5	TS=(nivolumab or "BMS 936558" or BMS936558 or "MDX 1106" or MDX 1106 or "ONO 4538" or ONO4538 or Opdivo or "AMP 514" or AMP514 o r MEDI0680 or "MEDI 0680" or atezolizumab or tecentriq or MPDL3280A or "MPDL 3280A" or durvalumab or imfinzi or "MEDI 4736" or MEDI4736 or avelumab or bavencio or MSB0010718C or "MSB 0010718C" or "BMS

	936559" or BMS936559 or MDX1105 or "MDX 1105" or cemiplimab or lib tayo or REGN2810 or "REGN 2810")
6	#1 OR #2 OR #3 OR #4 OR #5
7	TS=(cancer* or carcinom* or tumor* or tumour* or neoplas* or malignan* or metasta* or myeloma* or leukaemia* or leukemia* or lymphoma* or sarc oma* or melanoma*)
8	#7 AND #6
9	TS=((influenza* or flu or laiv) near/3 vaccin*)
10	#9 AND #8
	**Cochrane Library (Wiley). Date searched 01/21/2022**
1	((ipilimumab or "MDX CTLA 4" or Yervoy or "MDX 010" or MDX010 or tremelimumab or ticilimumab or "CP 675 206" or "CP 675206" or CP67520 or pembrolizumab or Keytruda or Lambrolizumab or "Merck 3475" or Merck3475 or "MK 3475" or MK3475 or "Sch 900475" or Sch900475)):ti,ab,kw[b]
2	(nivolumab or "BMS 936558" or BMS936558 or "MDX 1106" or MDX1106 or "ONO 4538" or ONO4538 or Opdivo or "AMP 514" or AMP514 or MEDI0680 or "MEDI 0680" or atezolizumab or Tecentriq or MPDL3280A or "MPDL 3280A" or durvalumab or imfinzi or "MEDI 4736" or MEDI4736 or avelumab or Bavencio or MSB0010718C or "MSB 0010718C" or "BMS 936559" or BMS936559 or MDX1105 or "MDX 1105" or cemiplimab or libtayo or REGN2810 or "REGN 2810"):ti,ab,kw
3	((immun* near/3 checkpoint near/3 (inhibitor* or modulator* or antibod* or block*))):ti,ab,kw[b]
4	(("cytotoxic T lymphocyte associated" near/3 "4") or "CTLA 4" or CTLA4):ti,ab,kw
5	(("Programmed Cell Death 1" or PD1 or "PD 1")):ti,ab,kw[b]
6	(("programmed death ligand 1" or "PD L1" or PDL1)):ti,ab,kw[b]
7	#1 or #2 or #3 or #4 or #5 or #6
8	((cancer* or carcinom* or tumor* or tumour* or neoplas* or malignan* or metasta* or myeloma* or leukaemia* or leukemia* or lymphoma* or sarcoma* or melanoma*)):ti,ab,kw[b]
9	#7 AND #8
10	((influenza* or flu or laiv) NEAR/3 vaccin*):ti,ab,kw[b]
11	#9 AND #10
	**Clinicaltrials.gov (National Library of Medicine). Date searched 01/21/2022**
1	checkpoint OR "Programmed Cell Death 1" OR PD1 OR "PD 1" OR "programmed death ligand 1" OR "PD L1" OR PDL1 OR "cytotoxic T lymphocyte associated" OR "CTLA 4" OR CTLA4 | cancer AND (influenza OR flu) AND vaccine

[a]Indexes=SCI-EXPANDED, SSCI, A&HCI, CPCI-S, CPCI-SSH, ESCI Timespan=1900-2022

[b]Word variations were also searched

Table S2. Risk of bias within studies assessed with the New-Castle Ottawa Scale[a]

	Selection				Comparability	Outcome			Overall score
**Study**	**Case definition**	**Case represen-tativeness**	**Control selection**	**Control definition**		**Exposure ascertain-ment**	**Non-response rate**		
Awadalla 2019 [19,20a]	✷	✷			✷✷	✷	✷		6
**Study**	**Exposed**	**Unexposed**	**Exposure**	**Outcome**		**Assessment**	**Follow-Up**	**Attrition**	
Bayle 2020 [9]	✷	N/A	✷		N/A	✷		N/A	3
Bersanelli 2018 [21,22]	✷	✷	✷	✷	✷✷	✷	✷	✷	9
Bersanelli 2021 [23-25]	✷	✷	✷	✷	✷✷	✷	✷	✷	9
Chong 2019 [26,27]		N/A	✷	✷	N/A	✷	✷	N/A	4
De Toma 2019 [28]		N/A	✷	✷	N/A	✷		N/A	3
Erickson 2021 [29]		✷	✷	✷		✷			4
Failing 2019 [30a]		✷	✷	✷	✷✷	✷	✷	✷	8
Gopalakrishnan 2018 [31]		✷	✷			✷	✷		4
Gwynn 2019 [32]		N/A	✷	✷	N/A	✷		N/A	3
Kanaloupitis 2017 [33]		N/A	✷	✷	N/A	✷		N/A	3
Keam 2019 [34,35]		N/A	✷	✷	N/A	✷		N/A	3
Laubli 2018 [36,37,40]		N/A	✷	✷		✷		N/A	3
Munoz Burgos 2018 [50]		✷	✷			✷			3
Reddy 2019 [38]		✷	✷		✷✷	✷			5
Roberts 2019 [39]		✷	✷			✷			3
Valachis 2021 [41]	✷	✷	✷	✷	✷✷	✷	✷	✷	9
Vutukuri 2021 [42]		✷	✷	✷		✷			4
Wijn 2018 [43]		✷	✷		✷	✷	✷		5

N/A, not applicable.
[a]For uncontrolled trials and case series non-relevant items (to evaluate a control group) were marked as N/A.

Table S3. Frequency of reported immune related adverse events in vaccinated and unvaccinated patients.

Study	Adverse event	Vaccinated (%)	Unvaccinated (%)
Awadalla 2019 [19,20a]	Hypophysitis<br>Hepatitis<br>Colitis<br>Dermatitis<br>Neurological<br>Gastritis<br>Pneumonitis<br>Cardiovascular death<br>Complete heart block<br>Cardiogenic shock<br>Cardiac arrest	4<br>4<br>8<br>0<br>4<br>0<br>12<br>36<br>9<br>9<br>9	7<br>9<br>9<br>8<br>13<br>4<br>36<br>72<br>19<br>20<br>17
Bayle 2020 [9]	Not specified	50	NA
Bersanelli 2018 [21,22]	NR	NR	NR
Bersanelli 2020 [23]	NR	NR	NR
Chong 2019 [26,27]	Endocrine (unspecified)<br>Pneumonitis<br>Colitis<br>Transaminitis	28<br>25<br>13<br>12	NA
De Toma 2019 [28]	Pneumonitis	4.8	NA
Erickson 2021 [29]	Cutaneous	68	40%
Failing 2019 [30]	Thyroiditis<br>Dermatitis<br>Colitis<br>Pneumonitis<br>Arthritis<br>Hepatitis<br>Adrenal insufficiency<br>Neuritis<br>Hypoglycemia<br>Hyperglycemia<br>Pancreatitis<br>Thrombocytopenia<br>Hearing loss	38.9<br>5.6<br>22.2<br>16.7<br>5.6<br>5.6<br>0<br>0<br>0<br>1<br>0<br>0<br>0	32.4<br>21.6<br>10.8<br>5.4<br>8.1<br>2.7<br>5.4<br>2.7<br>2.7<br>0<br>2.7<br>2.7<br>2.7
Gopalakrishnan 2018 [31]	Pneumonitis	17.2	37.2
Gwynn 2019 [32]	Diabetes<br>Nephritis<br>Hypothyroidism<br>Myalgia<br>Colitis<br>Rash	13<br>13<br>13<br>13<br>13<br>38	NA
Kanaloupitis 2017 [33]	Myalgia	4	NA
Keam 2019 [34,35]	Hypothyroidism<br>Pneumonitis<br>Inflammatory arthritis<br>Nephritis	2<br>2<br>2<br>2	NA
Laubli 2018 [36,37,40]	Rash<br>Arthritis<br>Colitis	13<br>13<br>8.7	NA

	Encephalitis<br>Hypothyroidism<br>Pneumonitis<br>Neuropathy	8.7<br>4.3<br>4.3<br>4.3	
Munoz Burgos 2018 [50]	Rash<br>Nephritis<br>Hypothyroidism<br>Neuritis<br>Liver dysfunction<br>Renal dysfunction<br>Psoriasis exacerbation	19<br>5<br>5<br>5<br>5<br>5<br>5	33<br>NR<br>NR<br>NR<br>NR<br>NR<br>NR
Reddy 2019 [38]	Colitis<br>Pneumonitis<br>Hepatitis	0<br>9<br>3	1<br>4<br>5
Roberts 2019 [39]	Not specified	4.4	10.8
Valachis 2021 [41]	Endocrine<br>Skin toxicity<br>Rheumatic<br>Hepatitis<br>Colitis<br>Pneumonitis<br>Neurologic<br>Renal toxicity<br>Other	11.5<br>10.6<br>4.7<br>5.5<br>3.4<br>4.3<br>1.7<br>1.3<br>4.3	6.9<br>3.4<br>10.3<br>0<br>6.9<br>3.4<br>6.9<br>3.4<br>6.9
Vutukuri 2021 [42]	Not specified	30.2	45
Wijn 2018 [43]	Endocrine (unspecified)<br>Pulmonary<br>Gastrointestinal<br>Hepatic<br>Arthritis<br>Neurological<br>Skin<br>Other	7<br>10<br>2<br>2<br>2<br>2<br>0<br>0	8<br>5<br>2<br>2<br>1<br>0<br>1<br>2

NR, not reported; NA, not applicable

[a]Awadalla et al 2019 reported rates of any type of irAEs in patients who already had myocarditis due to the immune checkpoint inhibitor.

Figure S1. Funnel plot for primary outcome (Egger test=0.55).

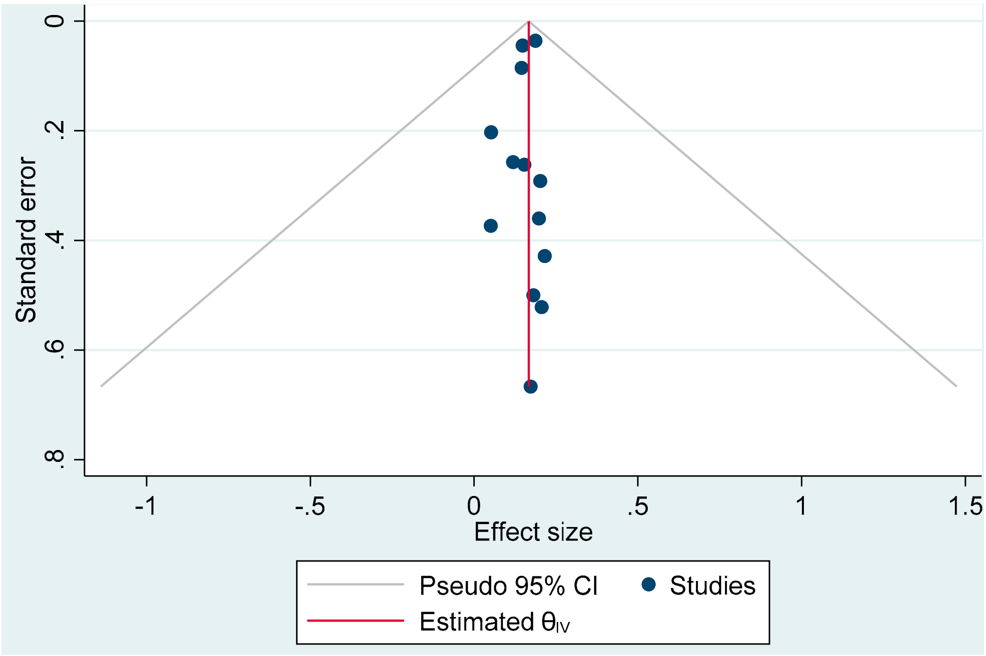

Table S4. Summary of findings table.

Outcomes	No. of participants<br>Studies<br>Follow-up (median time)	Quality of the evidence (GRADE)	Estimate (95% CI)	Comments
Proportion of irAEs	1334<br>17 studies (15 observational, 2 trials)<br>60 days to 31 months	⊕⊕⊖⊖<br>Low due to risk of bias and inconsistency[a,b]	Proportion 30%, 95% CI 22% to 40%	-
Risk of developing immune-related adverse events	2485<br>10 studies (10 observational)<br>107 days to 28 months	⊕⊕⊖⊖<br>Low due to risk of bias[a]	RR 0.90, 95% CI 0.72 to 1.1	-
Rates of vaccination among participants with irAEs	540<br>1 study (observational)<br>175 to 290 days	⊕⊖⊖⊖<br>Very low due to risk of bias, indirectness and publication bias[a,c,d]	OR 0.54 (95% CI 0.34 to 0.86)	-
Median time from vaccination to irAEs	555<br>4 studies (3 observational, 1 trial)<br>60 days to 519 days	⊕⊖⊖⊖<br>Very low due to risk of bias and inconsistency[a,b]	37 days (range 14-60) to 3.2 months (range 0-10.6)	-

[a]Observational studies without adjustment for confounding risks factors and uncontrolled case series. “In the GRADE approach to quality of evidence, observational studies without special strengths or important limitations provide low quality of evidence”[18]. However, for ethical reasons, no randomized controlled trials have been conducted to evaluate the benefits and harms of influenza vaccination in patients receiving ICIs.
[b]Inconsistency score higher than 50%.
[c]Surrogate outcome (indirectly measuring the probability of irAEs).
[d]Results from only one retrospective observational study.
